# Prognostic significances of PD-L1- and CTLA-4-positive T cells and positive correlations of immunosuppressive marker expression between cancer tissue and peripheral blood in patients with gastric cancer

**DOI:** 10.3389/fimmu.2023.1138743

**Published:** 2023-04-21

**Authors:** Kun Hee Lee, So Jung Kim, Jin Seok Woo, Seung Yoon Lee, Jooyeon Jhun, Jeonghyeon Moon, Yoon Ju Jung, Mi-La Cho, Kyo Young Song

**Affiliations:** ^1^ Rheumatism Research Center, Catholic Research Institute of Medical Science, College of Medicine, The Catholic University of Korea, Seoul, Republic of Korea; ^2^ Lab of Translational ImmunoMedicine, Catholic Research Institute of Medical Science, College of Medicine, The Catholic University of Korea, Seoul, Republic of Korea; ^3^ Department of Biomedicine and Health Sciences, College of Medicine, The Catholic University of Korea, Seoul, Republic of Korea; ^4^ Division of Gastrointestinal Surgery, Department of Surgery, Seoul St. Mary’s Hospital, College of Medicine, The Catholic University of Korea, Seoul, Republic of Korea; ^5^ Departments of Neurology and Immunobiology, Yale School of Medicine, New Haven, CT, United States; ^6^ Division of Gastrointestinal Surgery, Department of Surgery, Yeouido St. Mary’s Hospital, College of Medicine, The Catholic University of Korea, Seoul, Republic of Korea; ^7^ Department of Medical Life Sciences, College of Medicine, The Catholic University of Korea, Seoul, Republic of Korea

**Keywords:** gastric cancer, tumor microenvironment, programmed death-ligand-1, cytotoxic T lymphocyte antigen-4, interleukin-10

## Abstract

**Introduction:**

Although tumor, node, metastasis (TNM) staging has been used for prognostic assessment of gastric cancer (GC), the prognosis may vary among patients with the same TNM stage. Recently, the TNM-Immune (TNM-I) classification staging system has been used for prognostic assessment of colorectal cancer based on intra-tumor T-cell status, which is a superior prognostic factor compared with the American Joint Committee on Cancer staging manual. However, an immunoscoring system with prognostic significance for GC has not been established.

**Method:**

Here, we evaluated immune phenotypes in cancer and normal tissues, then examined correlations between tissues and peripheral blood. GC patients who underwent gastrectomy at Seoul St. Mary’s Hospital between February 2000 and May 2021 were included. We collected 43 peripheral blood samples preoperatively and a pair of gastric mucosal samples postoperatively, including normal and cancer mucosa, which did not influence tumor diagnosis and staging. Tissue microarray samples of GC were collected from 136 patients during surgery. We investigated correlations of immune phenotypes between tissues and peripheral blood using immunofluorescence imaging and flow cytometry, respectively. GC mucosa exhibited an increased number of CD4^+^ T cells, as well as increased expression levels of immunosuppressive markers (e.g., programmed death-ligand-1 [PD-L1], cytotoxic T lymphocyte antigen-4 [CTLA-4], and interleukin-10), in CD4+ T cells and non-T cells.

**Result:**

The expression levels of immunosuppressive markers were significantly increased in cancer tissues and peripheral blood mononuclear cells. In gastric mucosal tissues and peripheral blood of GC patients, similar immunosuppression phenotypes were observed, including increased numbers of PD-L1- and CTLA-4-positive T cells.

**Discussion:**

Therefore, peripheral blood analysis may be an important tool for prognostic assessment of GC patients.

## Introduction

Gastric cancer (GC) is one of the most common cancers in East Asia, which is ranks 5th in incidence and was the 4th leading cause of death among all solid cancers in South Korea excluding non-melanoma skin cancer in 2020 (1). In South Korea, new patients of gastric cancer (26,662 cases) ranked 4th (10.8%), followed by thyroid cancer (11.8%), lung cancer (11.7%), and colorectal cancer (11.2%), with a slight difference in 2020, according to the report of the Korea Central Cancer Registry (2, 3). In South Korea, early diagnosis of GC is common because esophagogastroduodenoscopy is widely performed for screening, and the proportion of patients with advanced GC (AGC) is decreasing (4). However, GC diagnosis and prognostic prediction can only be conducted using invasive methods, such as endoscopic biopsy. Although tumor markers (e.g., carcinoembryonic antigen and cancer antigen 19-9) are commonly used, they have limited utility in GC because of their low sensitivity and specificity (5, 6).

The Korean Practice guidelines for GC state that tumor, node, metastasis (TNM) staging is a useful indicator of cancer patient prognosis; treatment should be determined on the basis of the stage (7). Although TNM staging has been used for prognostic assessment of GC, the prognosis and clinical outcomes significantly vary among patients with the same TNM stage (8). The classification system provides limited prognostic information and does not predict the treatment response (9). Recently, the TNM-Immune (TNM-I) classification staging system has been used for prognostic assessment of colorectal cancer based on intra-tumor T-cell status, which is a superior prognostic factor compared with the American Joint Committee on Cancer staging manual (10).

Several recent studies have revealed relationships of immune-related markers with the treatment response, prognosis, and survival rate in GC treated with chemotherapy. The addition of molecular markers to TNM staging provides additional information regarding GC (11–13). Cancer progression depends on crosstalk between cancer cells and the immune system (14). GC characteristics (e.g., metastasis, treatment resistance, and disease recurrence) are associated with a tumor subpopulation known as GC stem cells (14). GC patients have reduced cancer suppression function in immune cells around cancer tissues. Honjo and Allison were awarded the 2018 Nobel Prize for their discovery of programmed death-ligand-1 (PD-L1) and cytotoxic T lymphocyte antigen-4 (CTLA-4), co-stimulatory factors that regulate cancer and autoimmune diseases (15, 16). Interleukin (IL)-10, which exhibits carcinogenic behavior, is a marker of GC and a potential therapeutic target (17). In the treatment of AGC patients, molecular markers are targeted *via* monoclonal antibodies, such as nivolumab and pembrolizumab; this constitutes a molecular approach for the treatment of AGC (18). Factors that decrease immune function (e.g., PD-L1, CTLA-4, and IL-10) are significantly increased in the immune and cancer cells in cancer tissues (17, 19–21). Immune cells activated or produced locally in gastric mucosa may reach systemic circulation and be detected in peripheral blood samples (22). However, the correlations and interactive effects of these cells in GC have not been elucidated.

In the present study, we evaluated differences in immune phenotypes between cancer and normal tissues, then examined correlations of immune phenotypes between GC tissues and peripheral blood.

## Materials and methods

### Study population

This study enrolled patients with gastric adenocarcinoma diagnosed preoperatively on endoscopic biopsy. All patients underwent conventional radical gastrectomy with curative intent, in accordance with the Korean Gastric Cancer Treatment Guidelines at Seoul St. Mary’s Hospital between February 2000 and May 2021. Patients with early GC (EGC) underwent D1+ lymph node dissection, whereas patients with locally advanced cancer underwent D2 or D2+ lymph node dissection. In total, 43 peripheral blood samples and gastric mucosal tissue samples were collected. Furthermore, a pair of gastric mucosal samples was obtained preoperatively, including normal and cancer mucosa, which did not influence tumor diagnosis and staging. Tissue microarray samples of GC were collected from 136 patients during surgery. The pathological stage of GC was classified in accordance with the criteria of the eighth American Joint Committee on Cancer. Patients with stage I and II disease were included in the EGC group, whereas patients with stage III disease were included in the AGC group. This study protocol was approved by the Institutional Review Board of the College of Medicine, Catholic University of Korea (KC20TISI0985). Patient records were anonymized before analysis.

### Intracellular staining and flow cytometry

Human peripheral blood mononuclear cells were isolated from blood samples of GC patients using Ficoll-Paque (GE Healthcare, Chicago, IL, USA), then stimulated with 25 ng/mL phorbol myristate acetate and 250 ng/mL ionomycin (Sigma-Aldrich, St. Louis, MO, USA) in the presence of GolgiStop (BD Biosciences, San Jose, CA, USA) for 4 h. Surface staining was performed with surface Alexa Fluor^®^ 700-conjugated anti-CD4^+^ (BD Pharmingen, Franklin Lakes, NJ, USA), allophycocyanin-C7-conjugated anti-CD8^+^ (BD Pharmingen), phycoerythrin-conjugated anti-CTLA-4, and fluorescein isothiocyanate-conjugated anti-PD-L1 (Biolegend, San Diego, CA, USA) antibodies. Samples were analyzed using FACSCalibur (BD Pharmingen) and a fluorescence-activated cell sorting instrument. Data were analyzed using FlowJo software (Tree Star, Ashland, OR, USA).

### Immunofluorescence analysis

Mucosa from GC patients was fixed in 10% formalin and embedded in paraffin. Paraffin-embedded sections were probed with anti-CD4^+^ (Novus Biologicals, Littleton, CO, USA), anti-CD8^+^ (Novus Biologicals), anti-PD-L1 (Invitrogen, Carlsbad, CA, USA), and anti-CTLA-4 (Invitrogen) primary antibodies at 4°C overnight. They were then stained with secondary antibodies conjugated with fluorescein isothiocyanate (Santa Cruz Biotechnology, Santa Cruz, CA, USA), allophycocyanin (Invitrogen), and phycoerythrin (Southern Biotech, Birmingham, AL, USA) at room temperature for 2 h. Nuclei were stained with 4,’6-diamidino-2-phenylindole (DAPI; Invitrogen). Immunofluorescence images were obtained using an LSM 700 confocal microscope (Zeiss, Oberkochen, Germany) at 200× magnification. Images were analyzed using ZEN 2 (blue edition) (Zeiss).

### Statistical analysis

Data are shown as means ± standard errors of the mean. Statistical analyses were performed using GraphPad Prism software (version 8; GraphPad Software, San Diego, CA, USA). Normally distributed continuous data were analyzed using Student’s *t-*test. Differences in means among groups were evaluated using one-way analysis of variance. *P* < 0.05 was considered indicative of statistical significance.

## Results

### Patient characteristics

The participants’ clinicopathological characteristics are shown in **Table 1**. The mean patient age was 59.2 years, and 68.4% of the participants were men. There were 47 and 89 patients with EGC (stage I and II) and AGC (stage III), respectively. There were significant differences between patients with EGC and AGC in terms of the extent of resection (subtotal gastrectomy, 85.1% and 55.1%, respectively; p = 0.001), Lauren classification subtype (intestinal type, 57.4% and 33.7%, respectively; p = 0.008), tumor size (4.2 ± 2.4 and 6.7 ± 2.8cm, respectively; p < 0.001), and positive lymph node ratio (0.04 ± 0.06 and 0.18 ± 0.13, respectively; p < 0.001). Lymphatic and neural invasion were significantly more common in AGC patients than in EGC patients (lymphatic invasion, 48.9% and 97.8%, respectively; p < 0.001; neural invasion, 17.0% and 67.4%, respectively; p < 0.001).

**Table 1:** Clinicopathologic characteristics of patients with gastric cancer according to pStages.

		pStage		
Characteristics	Total (n=136)	I, II (n=47)	III, IV (n=89)	*p*-value
Age, mean ± SD (yrs)	59.2 ± 11.0	58.9 ± 9.5	59.2 ± 11.8	0.823
Sex				0.267
male	93 (68.4%)	35 (74.5%)	58 (62.5%)	
female	45 (31.6%)	12 (25.5%)	31 (34.8%)	
Approach of surgery				0.585
Open	134 (98.5%)	47 (100%)	87 (97.8%)	
Laparoscopic	2 (1.5%)	0	2 (2.2%)	
Extent of resection				0.001
TG	47 (34.6%)	7 (14.9%)	40 (44.9%)	
STG	89 (65.4%)	40 (85.1%)	49 (55.1%)	
LN dissection				0.559
<D1+	25 (18.4%)	7 (14.9%)	18 (20.2%)	
>D2	110 (80.9%)	40 (85.1%)	70 (78.7%)	
others	1 (0.7%)	0	1 (1.1%)	
R0 resection	121 (89.0%)	45 (95.7%)	76 (85.4%)	0.067
Differentiation				0.127
Differentiated	49 (36.0%)	21 (44.7%)	28 (31.5%)	
Undifferentiated	87 (64.0%)	26 (55.3%)	61 (68.5%)	
Lauren classification				0.008
Intestinal	57 (41.9%)	27 (57.4%)	30 (33.7%)	
Diffuse/mixed	79 (58.1%)	20 (42.6%)	59 (66.3%)	
Tumor size (cm)	5.9 ± 2.9	4.2 ± 2.4	6.7 ± 2.8	<0.001
Retrieved LN (number)	42.6 ± 14.9	38.2 ± 13.4	45.0 ± 15.2	0.012
Positive LN ratio	0.14 ± 0.13	0.04 ± 0.06	0.18 ± 0.13	<0.001
pT				<0.001
1	22 (16.2%)	22 (46.8%)	0	
2	10 (7.4%)	7 (14.9%)	3 (3.4%)	
3	37 (27.2%)	17 (36.2%)	20 (22.5%)	
4	67 (49.3%)	1 (2.1%)	66 (74.2%)	
pN				<0.001
0	23 (16.9%)	23 (48.9%)	0	
1	33 (24.3%)	15 (31.9%)	18 (20.2%)	
2	30 (22.1%)	7 (14.9%)	23 (25.8%)	
3	48 (35.3%)	2 (4.3%)	48 (53.9%)	
Lymphatic invasion, yes	110 (80.9%)	23 (48.9%)	87 (97.8%)	<0.001
Venous invasion, yes	21 (15.4%)	4 (8.5%)	17 (19.1%)	0.195
Neural invasion, yes	68 (50.0%)	8 (17.0%)	60 (67.4%)	<0.001

SD, Standard deviation; TG, Total gastrectomy; STG, Subtotal gastrectomy; LN, Lymph node.

The English in this document has been checked by at least two professional editors, both native speakers of English. For a certificate, please see:

http://www.textcheck.com/certificate/5YClmH

### Analysis of peripheral blood and gastric mucosal samples from GC patients

**Figure 1:**
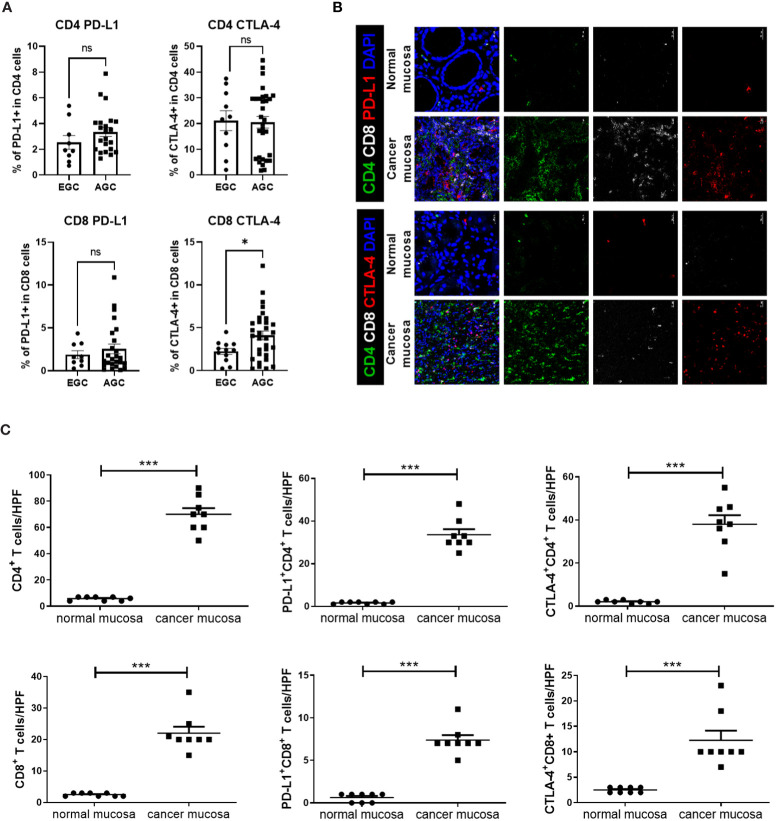
Expression levels of immunosuppressive markers, such as PD-L1 and CTLA-4, on T cells were higher in blood and cancer tissue from GC patients. Peripheral blood mononuclear cells from GC patients were stimulated with phorbol myristate acetate and ionomycin for 4 h, followed by GolgiStop for an additional 2 (h) Normal and cancer mucosa were harvested from GC patients, then stained with CD4+, CD8+, PD-L1, CTLA-4, and DAPI. **(A)** Bar graphs show percentages of PD-L1^+^ CD4^+^ T cells (top and left), CTLA-4^+^ CD4^+^ T cells (top and right), PD-L1^+^ CD8^+^ T cells (bottom and left), and CTLA-4^+^ CD8^+^ T cells (bottom and right) in peripheral blood mononuclear cells from early GC (EGC) and advanced GC (AGC) patients. **(B)** Representative confocal images showing PD-L1^+^ CD4^+^, CTLA-4^+^ CD4^+^, PD-L1^+^ CD8^+^, and CTLA-4^+^ CD8^+^ T cells in normal (n = 8) and mucosa (n = 8) mucosa. **(C)** Bar graphs show mean number of cells per high-power field (HPF) in normal and cancer mucosa. Scale bar = 20 μm. Data are means ± standard errors of the mean (*p < 0.05, ***p < 0.001).

Flow cytometry revealed higher expression levels of immunosuppressive markers, such as PD-L1 and CTLA-4, in CD4^+^ and CD8^+^ T cells from peripheral blood among AGC patients than among EGC patients, although a statistically significant difference was only observed for CTLA-4^+^ CD8^+^ T cells (**Figure 1A**). Immunofluorescence images showed higher numbers of CD4^+^ and CD8^+^ T cells in GC mucosal tissue than in normal mucosal tissue. Additionally, expression levels of immunosuppressive markers on CD4^+^ and CD8^+^ T cells were greater in cancer mucosa tissue than in normal mucosa tissue (**Figures 1B, C**).

### Analysis of GC mucosal tissue according to cancer stage

**Figure 2:**
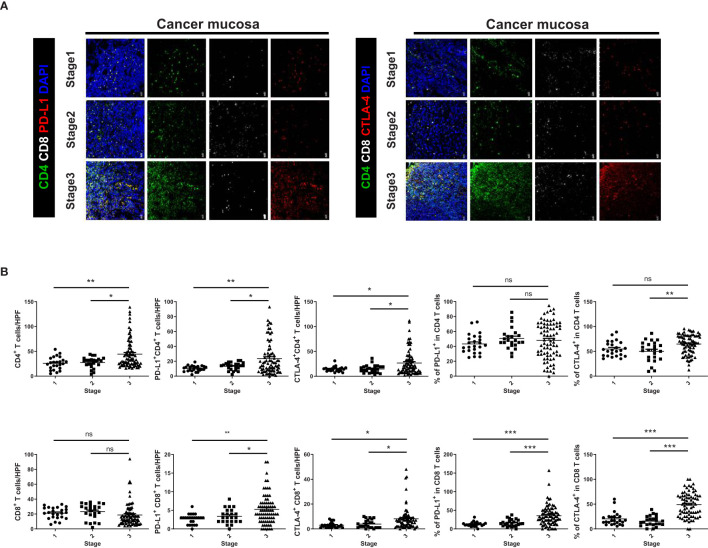
Expression levels of immunosuppressive markers on CD4^+^ and CD8^+^ T cells were increased in cancer tissue from GC patients with increasing TNM stage. **(A)** Confocal microscopic analysis of cancer mucosa from GC patients. Representative confocal images showing PD-L1^+^ CD4^+^, CTLA-4^+^ CD4^+^, PD-L1^+^ CD8^+^, and CTLA-4^+^ CD8^+^ T cells in cancer tissues. **(B)** Bar graphs show mean number of cells per HPF in cancer tissues according to cancer stage (Stages I–III, n = 24, = 23, and = 83, respectively). Scale bar = 20 μm. Data are means ± standard errors of the mean (*p < 0.05, **p < 0.01, ***p < 0.001).

Immunofluorescence images showed higher expression levels of immunosuppressive markers, such as PD-L1 and CTLA-4, in CD4^+^ and CD8^+^ T cells from cancer mucosa of GC patients as the cancer stage increased (**Figure 2A**). The proportion of CD4^+^ T cells was significantly greater in stage III cancer than in stages I or II, whereas there was no significant difference in the number of CD8^+^ T cells according to cancer stage. The numbers of PD-L1^+^ CD4^+^T, CTLA-4^+^ CD4^+^T, PD-L1^+^ CD8^+^ T, and CTLA-4^+^ CD8^+^ T cells increased as the cancer stage increased. The expression levels of immunosuppressive markers in CD4^+^ T cells increased with increasing CD4^+^ T cell infiltration into cancer mucosa. Therefore, the percentages of PD-L1 and CTLA-4 expression in CD4^+^ T cells did not differ according to cancer stage. The number of infiltrating CD8^+^ T cells in cancer mucosa did not significantly differ according to cancer stage; however, the levels of PD-L1 and CTLA-4 expression were increased in CD8^+^ T cells (**Figure 2B**). Our results suggest that immunosuppression in cancer mucosa increases with increasing cell number and increasing proportions of immunosuppressive marker-positive CD4^+^ and CD8^+^ cells, respectively.

### Correlations of immunosuppressive markers in CD4^+^ and CD8^+^ T cells from cancer tissue of GC patients

**Figure 3:**
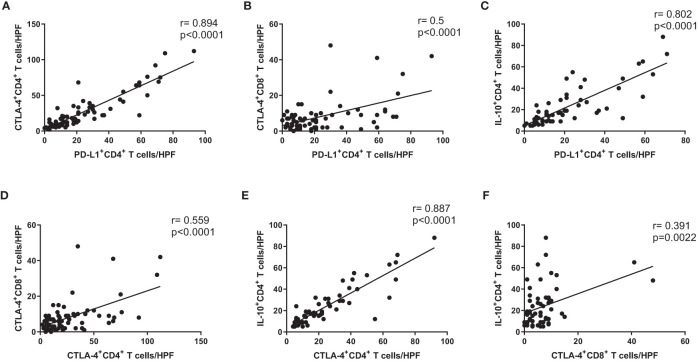
Expression levels of immunosuppressive markers on CD4^+^ and CD8^+^ T cells were correlated with each other in cancer tissue from GC patients. Correlation analysis of PD-L1^+^ CD4^+^ T cells with **(A)** CTLA-4^+^ CD4^+^ T cells, **(B)** CTLA-4^+^ CD8^+^ T cells, and **(C)** IL-10^+^ CD4^+^ T cells in cancer tissue from stage III cancer patients. Correlation analysis of CTLA-4^+^ CD4^+^ T cells with **(D)** CTLA-4^+^ CD8^+^ T cells and **(E)** IL-10^+^ CD4^+^ T cells in cancer tissue from stage III cancer patients. Correlation analysis of CTLA-4^+^ CD8^+^ T cells with **(F)** IL-10^+^ CD4^+^ T cells in cancer tissue from stage III cancer patients.

We investigated correlations of immunosuppressive markers (e.g., PD-L1, CTLA-4, and IL-10) in CD4^+^ and CD8^+^ T cells from cancer mucosa of GC patients. There were significant correlations involving the numbers of PD-L1^+^ CD4^+^ T cells/high-power field (HPF) and CTLA-4^+^ CD4^+^ T cells/HPF (**Figure 3A**), the number of PD-L1^+^ CD4^+^ T cells/HPF and CTLA-4^+^ CD8^+^ T cells/HPF (**Figure 3B**), the numbers of PD-L1^+^ CD4^+^ T cells/HPF and IL-10^+^ CD4^+^ T cells/HPF (**Figure 3C**), the numbers of CTLA-4^+^ CD4^+^ T cells/HPF and CTLA-4^+^ CD8^+^ T cells/HPF (**Figure 3D**), the numbers of CTLA-4^+^ CD4^+^ T cells/HPF and IL-10^+^ CD4^+^ T cells/HPF (**Figure 3E**), and the numbers of CTLA-4^+^ CD8^+^ T cells/HPF and IL-10^+^ CD4^+^ T cells/HPF (**Figure 3F**). These results showed that the numbers of immunosuppressive CD4^+^ and CD8^+^ T cells were correlated with each other in cancer mucosa from GC patients.

### Correlations of immunosuppressive markers in CD4^+^ T cells, CD8^+^ T cells, and macrophages from cancer tissue of GC patients

**Figure 4:**
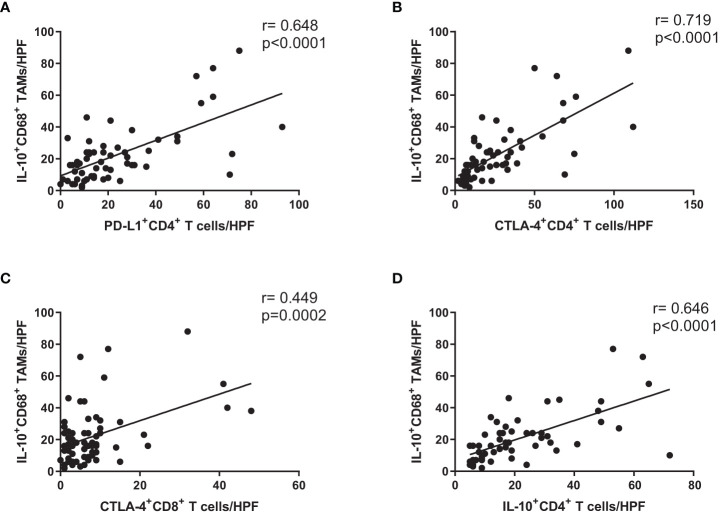
Expression levels of immunosuppressive markers on CD4^+^ and CD8^+^ T cells were correlated with number of IL-10-producing CD68^+^ macrophages in cancer tissue from GC patients. Correlation analysis of **(A)** PD-L1^+^ CD4^+^ T cells, **(B)** CTLA-4^+^ CD4^+^ T cells, **(C)** CTLA-4^+^ CD8^+^ T cells, and **(D)** IL-10^+^ CD4^+^ T cells with number of IL-10^+^ CD68^+^ TAMs in cancer tissue from stage III GC patients.

We evaluated IL-10-producing CD68^+^ tumor-associated macrophages (TAMs) in cancer tissue from GC patients. There were significant correlations involving the numbers of PD-L1^+^ CD4^+^ T cells/HPF and IL-10^+^ CD68^+^ TAMs/HPF (**Figure 4A**), the numbers of CTLA-4^+^ CD4^+^ T cells/HPF and IL-10^+^ CD68^+^ TAMs/HPF (**Figure 4B**), the numbers of CTLA-4^+^ CD8^+^ T cells/HPF and IL-10^+^ CD68^+^ TAMs/HPF (**Figure 4C**), and the numbers of IL-10^+^ CD4^+^ T cells/HPF and IL-10^+^ CD68^+^ TAMs/HPF (**Figure 4D**). These results showed that the numbers of immunosuppressive CD4^+^ and CD8^+^ T cells were also correlated with the numbers of IL-10-producing CD68^+^ TAMs in cancer mucosa from GC patients.

## Discussion

In this study, we evaluated whether immune cells (CD4^+^ and CD8^+^ T cells) and immunosuppressive markers (PD-L1, CTLA-4, and IL-10) were present in peripheral blood and cancer tissues from GC patients, then investigated whether those findings were correlated with each other. Several recent studies have revealed correlations of immunosuppressive markers with GC (22–24). Our results showed that the number of CTLA-4^+^ CD8^+^ T cells in peripheral blood was significantly greater among AGC patients than among EGC patients. The numbers of CD4^+^ and CD8^+^ T cells, as well as the expression levels of their immunosuppressive markers, were greater in cancer mucosa than in normal mucosa. There were also significant differences among cancer stages. The number of CD4^+^ T cells was greater in stage III than in other stages, whereas the number of CD8^+^ T cells did not differ according to cancer stage. The numbers of PD-L1^+^ CD4^+^ T, CTLA-4^+^ CD4^+^ T, PD-L1^+^ CD8^+^ T, and CTLA-4^+^ CD8^+^ T cells increased with increasing disease stage. The expression levels of immunosuppressive markers in CD4^+^ T cells from cancer mucosa increased with increasing cancer stage. Therefore, the percentages of PD-L1- and CTLA-4-positive CD4^+^ T cells did not differ according to cancer stage. In contrast, the infiltration of CD8^+^ T cells did not significantly differ with cancer progression; however, the percentages of PD-L1- and CTLA-positive CD8^+^ T cells were increased. Therefore, the levels of immunosuppressive markers in CD8^+^ T cells increased with cancer progression. Our results suggest that the levels of immunosuppressive markers in immune cells are closely related to GC, and the distribution patterns of circulating markers in GC tissues are correlated with the patterns of markers in peripheral blood. Although it is unclear whether immunosuppression is a cause or consequence of GC, our results showed that peripheral blood sampling may be useful in prognostic prediction for GC patients.

There are increasing numbers of immunological and molecular studies focused on GC. Sánchez-Zauco et al. (25) performed a comparative analysis of circulating markers between GC patients and healthy controls. *Helicobacter pylori* activates a specific signaling cascade, thereby inducing several cytokines and chemokines that lead to GC (26–28). In a study of blood samples collected from patients before surgery, interferon-γ and IL-10 were identified as diagnostic markers for EGC; IL-1β, IL-8, and macrophage chemotactic protein-1 were identified as diagnostic markers for AGC. In the present study, we also analyzed markers present in the cancer mucosa, which were excluded from analysis in previous studies. The strength of our study is that we identified a correlation between immune markers in cancer tissue and peripheral blood from GC patients.

This study had some limitations. First, it was a single-center study with a small sample size. Moreover, disease biomarkers are influenced by ethnicity, country, environment, and lifestyle (29–32). Thus, it is difficult to generalize our results to other institutions or countries. Therefore, future studies should evaluate the utilities of biomarkers for various ethnicities, countries, and cultures. Second, despite substantial efforts to identify cancer biomarkers over the past 15 years, only a few markers have been identified with utility in cancer diagnosis and monitoring (33). Because of variations in molecular characteristics, the utility of a candidate biomarker cannot be determined. Mechanisms underlying the roles of specific markers may differ according to cancer type and tumor microenvironment. Therefore, further studies are needed to explore molecular mechanisms that underlie biomarkers and their effects. In conclusion, there were similar immunosuppression phenotypes in gastric mucosal tissues and peripheral blood from GC patients. We found correlations between disease severity and the expression levels of immunosuppressive markers. These findings suggest that peripheral blood analysis can be used as a prognostic tool and facilitate the development of anti-cancer therapy directed against immune cells.

## Data availability statement

The original contributions presented in the study are included in the article/supplementary material. Further inquiries can be directed to the corresponding authors.

## Ethics statement

The studies involving human participants were reviewed and approved by the institutional review board of the College of Medicine, Catholic University of Korea (KC20TISI0985). The patients/participants provided their written informed consent to participate in this study.

## Author contributions

KHL, M-LC and KYS conceived and designed the study. KHL, SJK, JSW, JM and YJJ wrote the manuscript and performed the data analysis. KHL, SJK, JSW, SYL, and JYJ were responsible for data collection and reviewing the data analysis. M-LC and KYS reviewed the manuscript and provided feedback. All authors discussed the results and contributed to the final version of the manuscript.
